# Using Real-Time Dashboards to Monitor the Impact of Disruptive Events on Real Estate Market. Case of COVID-19 Pandemic in Australia

**DOI:** 10.1007/s43762-022-00044-z

**Published:** 2022-06-10

**Authors:** Balamurugan Soundararaj, Christopher Pettit, Oliver Lock

**Affiliations:** City Futures Research Centre, UNSW, Sydney, Australia

**Keywords:** Dashboards, Real-Estate Market, COVID19, Disasters, PropTech

## Abstract

Real estate markets are complex both in terms of structure and dynamics: they are both influenced by and influence almost all aspects of the economy and are equally vulnerable to the shocks experienced by the broader economy. Therefore, understanding the extent and nature of the impact of large-scale disruptive events such as natural disasters and economic financial downturns on the real estate market is crucial to policy makers and market stakeholders. In addition to anticipating and preparing for long-term effects, it has become imperative for stakeholders to monitor and manage the short-term effects as well due to the emergence of ‘PropTech’ and ‘platform real estate’. In this work, we explore the use of online, real-time dashboards which have been used extensively in the context of urban management, policymaking, citizen engagement and disaster response as an appropriate tool for the purpose of monitoring real estate markets. We describe the process of designing, building, and maintaining an operational dashboard for monitoring the residential real estate market in Australia during the COVID-19 pandemic in 2020. We detail the techniques and methods used in creating the dashboard and critically evaluate their feasibility and usefulness. Finally, we identify the major challenges in the process, such as the spatial and temporal availability and veracity of the real estate market data, and we identify possible avenues for consistent, high-quality data; methodology; and outputs for further research.

## Introduction

Real estate is one of the most important sectors in the economic system. Because of their central role in the economy, real estate markets tend to be large, complex, and nonlinear systems that are not only influenced by a myriad of external factors but also influence them directly and indirectly (Yunus et al. 2012). Due to the interdependencies between various sectors of the economy, real estate markets are vulnerable to sudden shocks and disruptions that happen in other sectors. These disruptions could be short-term events such as large-scale natural disasters (Bin and Kruse 2006; Park et al. 2019), or economic and financial downturns (Kallakmaa-Kapsta 2005) or they could be long-term changes such as increased terrorism or violence (Del Giudice et al. 2020) and sea-level rise (Bernstein et al. 2019). Therefore, the stakeholders of real estate markets need to devise and employ tools and techniques to measure and monitor the movements of the market quickly and easily, thus improving their risk management capabilities in the event of a major disruption (Park et al. 2019). Though risk management in real estate markets is usually associated with long-term financial risks and preparedness against natural disasters, in recent decades this has changed significantly due to the emergence of ‘platform real estate’ (Shaw 2020), where property technology (‘PropTech’) players constantly compete and innovate to make commodities on the market for real estate space and real estate assets smaller, more accessible, transparent, manageable and liquid. For example, Airbnb has transformed the traditional market for space in the hospitality and short-term renting industries (Chang 2020), and innovative real estate investment trusts (REITs) have made investments in real estate more liquid and efficient (Yunus et al. 2012). Hence, the stakeholders in real estate markets need to measure, track, and monitor the market and respond with decisions on almost a real-time basis in addition to traditional long-term preparedness.

Meanwhile, the pursuit to gather, analyse, visualise, and advise on the performance of large, nonlinear and complex urban systems has resulted in numerous online, real-time city dashboards (Jing et al. 2019). The accelerating development and adoption of these dashboards has been fuelled by the unprecedented volume and speed of data generated by urban systems under the ‘smart city’ umbrella where cutting edge Information Communications Technologies are integrated and employed in the study and management of Cities (Batty et al. 2012). This trend has been exacerbated by the increasing number of Internet of Things devices, such as sensors that stream massive amounts of data openly and regularly (Batty 2013; Batty 2015). Coupled with data generated by social media, these devices have enabled dashboards to abstract and report the state of the urban systems in real-time (Matheus et al. 2020). Many dashboards have been built and used extensively for information dissemination, citizen engagement and participation, urban management, public policy and decision-making, and more (Jing et al. 2019). In a command and control centre setting, real-time operational dashboards that provide descriptive measurements and statuses using indicators have been regularly utilised to manage natural disasters such as fires, flooding and earthquakes (Lock et al. 2020). During the COVID-19 pandemic, many dashboards have been released (Dong et al. 2020) to track the pandemic and monitor its effect on other sectors, such as industry, economy and society (Pellert et al. 2020; Marvel et al. 2021; Gualtieri et al. 2020). There is an evident need to understand the complex real estate market in real-time. Real-time operational city dashboards are capable and popular and could be used to monitor and understand the impact of sudden disruptions on real estate markets, thus providing value to stakeholders. However, there is a lack of prior literature in linking the two.

This work aims to fill this gap by systematically documenting the process of conceptualising, designing, building, deploying, and maintaining an operational dashboard linking the spread of COVID-19 in Australia with corresponding changes in the residential real estate market. The paper begins by identifying the possible data sources that can provide a near-real-time insight into the status of the residential real estate market and describing the implementation of a data-processing pipeline for data collection. The paper explains how the collected datasets were cleaned, filtered, aggregated and visualised on a single-page, auto-refreshing, interactive and responsive dashboard built on the RShiny platform. This dashboard was then deployed online using a micro-services architecture. The paper discusses the novel insights that were derived from the dashboard and reflects on their utility. There are pitfalls associated with dashboards, such as oversimplification, isolation from the broader context, uncertainties and biases in the data and analysis (Kitchin et al. 2015a). Therefore, the paper also critically discusses the challenges faced by the dashboard, such as the lack of reliable data sources, uncertain veracity of the existing data sources both spatially and temporally and the limited impact of COVID-19 in Australia in general. Finally, the paper formally measures the inferences derived from visual analytics to substantiate the utility of such a dashboard in the real estate market context.

## Literature

The literature related to the use of real-time dashboards in the real estate market can be broadly classified into three topics:

- The origin, evolution, purpose and application of city dashboards in the field of urban analytics along with the challenges encountered in creating, using, and maintaining them;
- The use of the experience, technologies, and techniques gained from city dashboards to monitor real estate markets; and
- The events that affect the real estate market (e.g., natural disasters and market cycles) and their impact on the real estate market, with a specific focus on the impact of the COVID-19 pandemic on markets around the world.

***A brief history and overview of city dashboards***

From the physical artifacts which gave a simplified status of complex machinery, dashboards have developed to become toolkits of urban planners, managers and policymakers to understand and manage the complex system of cities resulting in numerous city dashboards in the fields of research, industry and government. From the modest beginnings in CompStat in 1994, making city dashboards has rapidly developed in to a field with dedicated international standards in 2014 (Jing et al. 2019) leading to the notable city dashboards for various cities such as London (Gray et al. 2016), Dublin (McArdle and Kitchin 2016) and Sydney (Pettit et al. 2017).

City dashboards can be large classified into three types based on their purpose:

1. Operational dashboards, which inform the users about the status of the city;
2. Analytical dashboards, which derive inferences and diagnoses the system; and
3. Strategic dashboards, which predict future outcomes and provide guidance for decision-making (Jing et al. 2019).

There can be numerous subtypes of these dashboards based on their purpose, audience, features, layouts, refresh frequency and more (Sarikaya et al. 2018). Dashboards typically consist of two major components: indicators and analytical models. Indicators are the meaningful, comparable, understandable metrics derived from the data; they provide information about the underlying system. Designing the indicators is a major part of dashboard design: they can be simple or composite, static or real-time, and they can also be inputs for other indicators (Kitchin et al. 2015a). Analytical models are the logic that generate or transform the data and indicators; these are used for further inferences relevant to analytical and strategic dashboards. Apart from these components, successful dashboard design also involves careful consideration of the architecture, workflow, and visual design; this consideration must be informed by the purpose (Stehle and Kitchin 2020) and intended users (Young et al. 2021).

City dashboard development is an interdisciplinary field between urban planning, management, information technology and data science. It provides immense public value, such as improving the transparency and efficiency of the urban planning process and encouraging public engagement (Kourtit and Nijkamp 2018). Nonetheless, dashboards also have numerous potential issues in terms of epistemology of the research, scope and access of data sources, veracity and validity of the data, usability, literacy of the users and the ethics of using the dashboards (Kitchin and McArdle 2016). There are also practical problems, such as oversimplification, incorrect interpretation and confusion of outcome (Matheus et al. 2020). Moreover, these dashboards are complex socio-technical assemblages that influence the system they are set to mirror (Kitchin et al. 2016). Rather than being a linear process, the construction of these dashboards is a circular, continuous endeavour (Bellini et al. 2018) to improve their usage (Balletto et al. 2018).

***Use of dashboards to monitor real estate market***

The information technology revolution, which digitised cities and led to an explosion of free and open data, fuelled the rise of city dashboards. A similar rapid and significant paradigm shift is due to occur in the real estate sector in this decade. Shaw (2020) argues that the recent surge of innovation in PropTech is revolutionising the real estate market with the digitisation and democratisation of spatial and transactional data on what has been traditionally a marketplace for transacting physical space and assets. Along with technological improvements (e.g., blockchain, AI and machine learning), this has increased the efficiency of ownership models, transactions, analytics and strategies for investment. As a result, the real estate market and price discovery within it are significantly more open and transparent than in previous decades, and the decisions taken by stakeholders are now more data-driven than they were in the past.

In this context, Munawar et al. (2020) hypothesise that the real estate market of the big data era will become ‘smart real estate’ akin to ‘smart cites’. The quality of information and analytics provided by this smart real estate will bring great value to stakeholders by enabling personalisation to customers, accurate valuation to lenders, better management and transactional ease to owners, better fraud detection and taxation to the government, and flexibility and risk reduction to investors. In such a scenario real estate dashboards could play a similar role to city dashboards today. Although there have been few attempts for facilities management and public real estate management (Ladu 2020), there is no substantial research has investigated the use of dashboards in the context of real estate markets. There are very few online, real-time dashboards that monitor the market continuously; most of them are built from the investment portfolio management perspective (Curto and Fregonara 2019).

***Impact of disasters and disruptive events on the real estate market***

Large-scale disasters directly affect the real estate market by affecting liquidity, impacting fear and risk perception, increasing the cost of supply, and reducing demand. They also indirectly affect the market through changes in mobility, size/velocity/cycles of the overall economy and monetary or fiscal policy response to these changes. Although not extensive, there are prior studies that analyse and quantify the impact of such events on real estate markets. Research into flood-affected markets in United States (Bin and Kruse 2006; Simmons et al. 2002; Tobin and Montz 1988) shows that the increased risk perception after such an incident usually reduces demand and causes a premium for lending unless it is offset by government policy. In some cases, such as markets affected by rising sea levels, the impact could be up to 7% of the property prices (Bernstein et al. 2019). While studies on the Hong Kong real estate market after the SARS epidemic found only a marginal impact and a quick recovery, studies on U.S. cities after terrorist incidents found an increase in renting activity compared to buying (Del Giudice et al. 2020). It has also been shown that such incidents—along with government interventions like rent and mortgage reliefs or insurance subsidies—can disrupt overall market cycles in the economy (Morgan 2007).

Early studies suggest on the ongoing COVID19 pandemic and its impact show that almost all aspects of everyone’s socio-economic life of have been affected (Nicola et al. 2020; Gualtieri et al. 2020). Moreover, the reduction in mobility and the deceleration of economic activity (Maliszewska et al. 2020) have disproportionately affected the poor and vulnerable (Egede and Walker 2020; Bonaccorsi et al. 2020; Chang et al. 2021). This affects the real estate market as this segment of the population represents a sizeable portion of renters and vulnerable mortgage holders (Goodman and Magder 2020). Any significant impact on this segment can snowball out into the whole market.

In a study of the real estate market of the Campania region of Italy, Del Giudice et al. (2020) have found that the pandemic has caused the housing prices to drop by 4.16% and 6.49% in the short and medium term respectively. Ling et al. (2020) have examined the impact of the pandemic on the commercial real estate markets in USA and have found a similar negative effect, which has been moderated by government intervention. Studies on the hospitality industry in China (Hao et al. 2020) during the pandemic show that the whole industry has experienced a crash in prices and occupancy across the board, with mid-range offerings being the most affected. While the price of cheaper offerings bounced back, luxury offerings remained relatively unaffected. Showing the possibility of different impacts of disasters on different sections of the real estate market.

## Methodology

### Data collection

As shown by the literature survey in Section 2.0.3, disasters such as COVID19 affect the property market directly and indirectly through various factors. In order to build an operational dashboard, we set out to survey of data sources on four broad areas.

- Spread and severity of COVID-19 in Australia,
- Performance of the property market,
- Broader socio-economic context and
- Sentiment of the stakeholders and public.

The range of sources that were considered are detailed in Table 1. In addition to the traditional data sources, in the wake of the outbreak and widespread media attention, non-traditional sources such as Google, Apple and AirDNA emerged to show impact on their users which were also considered. It is important to note that, in a time critical exercise such as this, the availability of data influenced the viability and design of the dashboard as much as the formal data collection process.

**Table 1:** Data sources considered for understanding aspects of COVID-19 and Australian property market

Aspect	Data Sources
Spread of COVID-19	Johns Hopkins University dashboard
	Federal government websites on COVID-19
	State governments’ websites on COVID-19
Real Estate Market	Market monitors - CoreLogic, Domain etc.
	Government agency data sources - Valuer General, Rental Bond Board, etc.
	Commercial data providers - AirDNA, Open Airbnb
Economy and Activity	Transportation data from government departments
	Commercial application data such as Google Maps, Citymapper and Apple Maps etc.
	Economic reports from the Reserve Bank of Australia
Public Sentiment	Internet search trends from search engines such as Google
	Social media posts such as Twitter, Facebook, etc.

#### Spread and severity of COVID-19 in Australia

Being the most significant event in 2020, the data sources regarding the spread of COVID-19 pandemic were abundant such as WHO, National and State government agencies, technology companies, news agencies and even volunteer run projects. For this research, we considered the Johns Hopkins University (JHU) School of Public Health’s dashboard and the associated data repository, the Australian Government’s website, and the websites of state governments as feasible data sources. For the overall Australian COVID-19 data, we selected the JHU’s repository since the data in the repository are derived from government sources, cleaned, stored in a format that is easy to access and updated daily. This repository provided us with the number of cases, fatalities, and recoveries for Australia at national and state level. More granular level of data, such as local government areas (LGA) or local health districts (LHD) were gathered from the websites of individual states.

There were issues with the veracity of the data where the content and format were different and often changed with time between sources. For example, Victoria released data at LGA level while Queensland released them LHD level and New South Wales released both, while the rest of the states did not release data at such granularity. JHU data were published data as CSVs while other sources were in various formats such as HTML, PowerBI, Tableau etc. None of sources released granular geographic information on COVID19 cases to protect the privacy of the people involved.

#### Performance of the property market

Although various property market monitors collect and disseminate up-to-date, near-real-time data on property sales and valuations across the country, these datasets are not openly accessible due to their significant commercial value. Out of the publicly available open data, we chose to use CoreLogic’s House Value Index and domain.com.au’s reports on residential property auctions. CoreLogic’s index is a patented and proprietary product which is updated daily. It covers Australia’s major cities and shows the capital growth in the value of residential properties combining data on property attributes and transactions along with data from government agencies. It acts as a benchmark for comparing market changes across the country. Domain.com reports the statistics on the result of residential property auctions across major cities in Australia every week such as total number, clearance rate, total value and median price. Although this dataset is updated weekly and is biased towards more expensive properties, it provides a way to monitor, track and understand the movements of the market with more granularity compared to a composite index. To understand the movements in the securitised property market, we used the daily value of Standard & Poor index on real estate funds (XRE) from Australian Stock Exchange.

The State government agencies such as rental board, valuer general etc. release data on rental rates and property transactions regularly but they are often 2 to 6 months old and not suitable for monitoring the market in real-time. While the openly available data on Airbnb listings are updated infrequently, the proprietary and weekly AirDNA data is released as a tableau dashboard, discouraging further use. The availability of data across regions in Australia is inconsistent as well. For example, Cities such as Hobart and Darwin are covered in CoreLogic data but not on the Domain reports while the COVID-19 data are usually at the state or LGA level.

#### Broader socio-economic context

We tried to quantify the impact of COVID-19 on the broader economy in two ways: the reduction in physical activity and the national changes in economic and financial activity. Lock-downs caused by the COVID-19 outbreak resulted in reductions in physical activity, measurable with data from both the transport authorities and private companies such as Google, Apple and Citymapper. The transport authority datasets posed similar limitations to those we encountered with the property data: updates were infrequent, especially exacerbated by the lock-downs. Since data from Google and Apple were released as portable documents (pdf) and proved challenging for use as data sources, the index provided by Citymapper was collected and used for this research. This index shows the transport activity within various cities across the world, estimated from the use of the mobile application. Although the data source was updated regularly, it was only available for two cities in Australia: Sydney and Melbourne. Like data from every other government agency, the economic data from the Reserve Bank of Australia (RBA) were updated quite infrequently. For example, the datasets on mortgages and spending were updated quarterly, making them unsuitable for use with this dashboard.

#### Sentiment of the stakeholders and public

The pandemic has affected the property market through consumer and investor sentiments such as fear, uncertainty, and risk perception. Social media was identified as one of the best data sources for quantifying these sentiments and Twitter was selected as it supports granular, large-scale data collection in real-time. Although the twitter search API provides with much more targeted approach for collecting tweets, the API is limited in the total number of tweets it can return. The initial data collection also showed that the tweets focussing on just property and real-estate markets in Australia are extremely limited compared to more general tweets. For example, on between Dec 2019 and April 2020, there were only 2300 tweets relating just to property market while there were 175,000 tweets relating to COVID19. Keeping these in mind we switched to the streaming API which gives more comprehensive set of data. Moreover, it is also noted that using the streaming API with broad set of search terms gives a better pulse of the general market sentiment and thus a better comparison with COVID19 infection numbers especially in daily time intervals. An exhaustive list of 336 keywords was created by combining the variations of words ‘COVID-19’, ‘corona’, ‘property’, ‘real-estate’ and names of cities states across Australia. Tweets containing these terms (e.g. ‘realestatesydney’, ‘covid-19 AU’, ‘covid19QLD’) were collected and streamed into a database. An average of 56,000 tweets per day and a total of 11.6 million tweets were collected between April 2020 and Oct 2020. The trends in terms used in Google were also collected as a proxy for the relative importance of the property market and COVID-19 in the public discussion over time and in relation to each other. The daily trends for the search terms ‘COVID-19’ and ‘real estate’ were collected for Australia. This allows us to capture the broader contextual changes such as policies and rules in the country. It is important to note that data from Twitter has significant biases and noise in terms of the users, coverage and posts. Similarly, the proprietary Google Search trends data are comparative measures and need to be combined carefully with other absolute data.

### Data processing

#### Cleaning and storing the data

**Fig. 1:**
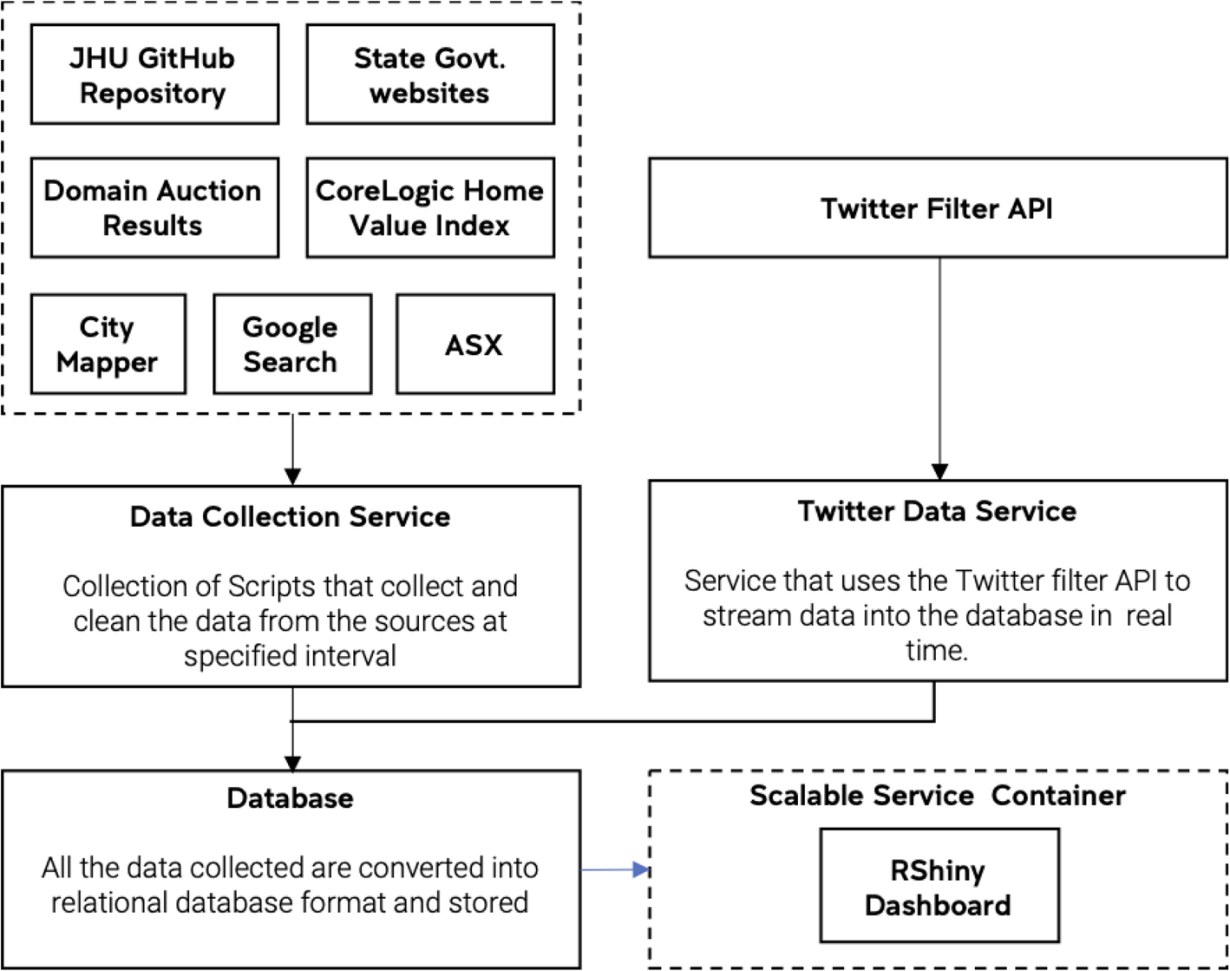
Overall System Diagram of the Dashboard

Some of the datasets collected were indices that were already processed and cleaned before publication; these did not need any further processing. The rest (weekly auction statistics from Domain, LGA-level COVID-19 statistics and Twitter data) needed a customised pipeline for collecting, cleaning and processing data before use. These datasets could be deemed to be ‘medium’ in size since they exhibit large amounts of variety and veracity but limited volume and velocity (Soundararaj et al. 2019). These datasets were cleaned and processed using appropriate data pipelines constructed from various web-scraping tools and Unix command-line utilities. The primary data collection tools used were curl, pup and jq. For datasets which are not in plain HTML format and need user interaction a combination of python, selenium library and chrome web driver was used. These pipelines were deployed as containers and deployed to the Amazon Web Services (AWS) as either a regular process or an ‘always on’ service. The data scraped by the pipeline was sent into a PostgreSQL database with three main tables - covid19, propertymarket and twitter and few minor tables regarding the economic indicators such as ASX 200 indices and mobility index. Figure 1 illustrates the overall design of this data collection pipeline.

#### Designing and implementing indicators

A total of 22 indicators and summary statistics were produced from the collected data. These indicators, their geographic extent and the source from which they were derived are shown in Table 2.

**Table 2:** Indicators, Data Sources and Processing of the Data

Indicator	Level	Data Source
COVID-19 Total Cases	National	JHU Repository
COVID-19 Total Cases (Daily)	National	JHU Repository
COVID-19 Recoveries	National	JHU Repository
COVID-19 Deaths	National	JHU Repository
COVID-19 Total Cases	State	JHU Repository
COVID-19 Total Cases	LGA	Government Websites
Auction Value	National	Domain
Auction Value (Annual Change)	National	Domain
Auction Value (Daily)	National	Domain
Auction Value	Cities	Domain
Auction Value (Annual Change)	Cities	Domain
Clearance Rate	Cities	Domain
Clearance Rate (Annual Change)	Cities	Domain
Auction Value (Weekly)	Cities	Domain
Clearance Rate (Weekly)	Cities	Domain
Median Price (Weekly)	Cities	Domain
House Value Index (Daily)	Cities	CoreLogic
Twitter Sentiment (15 mins)	National	Twitter
Mobility Index	Cities	Citymapper
ASX 200 XRE	Sydney	ASX
Google trends (realestate)	National	Google
Google trends (COVID-19)	National	Google

**Fig. 2:**
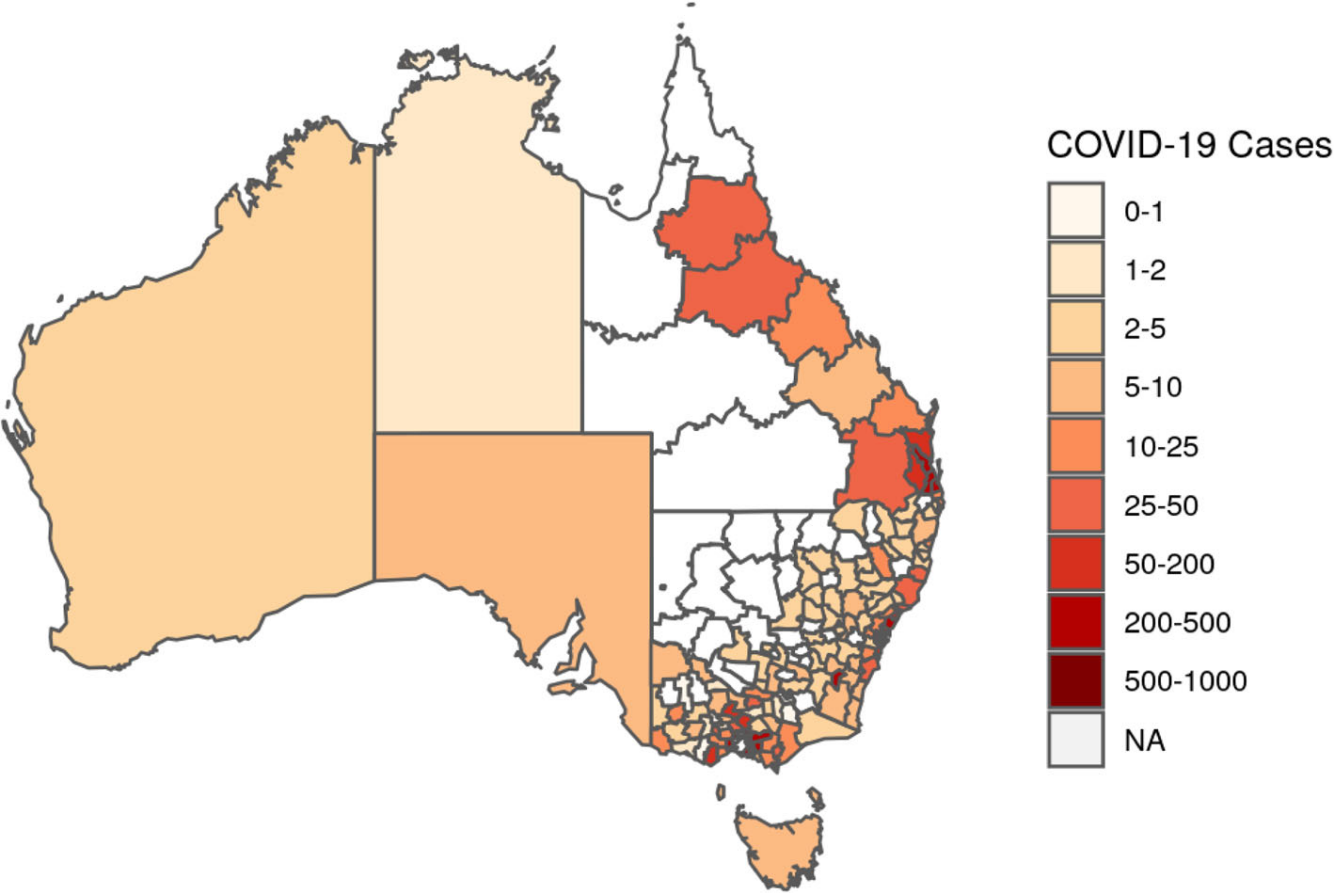
Distribution of Total COVID-19 Cases at LGA Level Across Australia

Across the COVID19 related indicators, the summary statistics were plotted as text, the daily totals were plotted as a line chart showing the change over time, and LGA-level active case numbers were depicted as static or interactive maps. A line chart of the daily total cases at the national level was also created for comparison with other national indices. It is important to note that the map (Fig. 2) is a composite which shows LHD areas in Queensland, LGA areas in New South Wales and Victoria and average cases per LGA for states without granular data.

In property market indicators, since domain research reports were not published in certain weeks, the gaps were linearly interpolated. Similar to the COVID-19 cases, the weekly number of auctions across Australia was created for comparison with other national indices. The performance indicators (total value of auction sales, clearance rates and median price for major cities) and the house value index were depicted as line charts showing change since January 2020. The House Value Index from CoreLogic for major cities was also used as an indicator. Though the House Value Index does not change substantially daily, the marginal daily changes were averaged on a 20-day rolling window and annualised (assuming 250 trading days) for comparison with returns from the equity market. This shows the daily momentum of the market in terms of the value of houses across the major markets, similar to the stock market. For further understanding, broader socio-economic context indicators (Citymapper Mobility Index for Sydney and Melbourne, Google trends for ‘real estate’ and ‘covid-19’, ASX 200 XRE, etc.) were also created and depicted as line charts showing changes since January 2020. The collected ‘tweets’ were aggregated into an indicator showing the Twitter sentiment index (defined as the difference between the number of positive and negative words in the tweets at a given 15-minute interval). First, the collected tweets were converted to plain text and filtered to remove special characters and re-tweets. They were then tokenised to generate a list of words. These words were then classified as positive, negative, and neutral using the ‘Bing’ lexicon; summarised to give the number of words in each sentiment; and subsequently used to provide the Twitter sentiment index for that interval. This sentiment index was then shown as a diverging bar chart at 15 minute intervals for the last 7 days.

#### Dashboard design and deployment

The overall COVID-19 Property Dashboard was built by combining the above indicators using the ‘Shiny’ library. The library is part of the R statistical programming language and handles both the front and back ends of the dashboard.

At the back end, datasets are queried from PostgreSQL, analysed in R and appropriate charts are generated before handing over to the front end. Since the query process is often the longest step, to optimise the performance of the dashboard, the queries are cached every hour. Similar to the data collection services, this ‘Shiny’ dashboard is also containerised and deployed in AWS ECS as an auto-scaling service which can increase or decrease its capacity in line with demand.

**Fig. 3:**
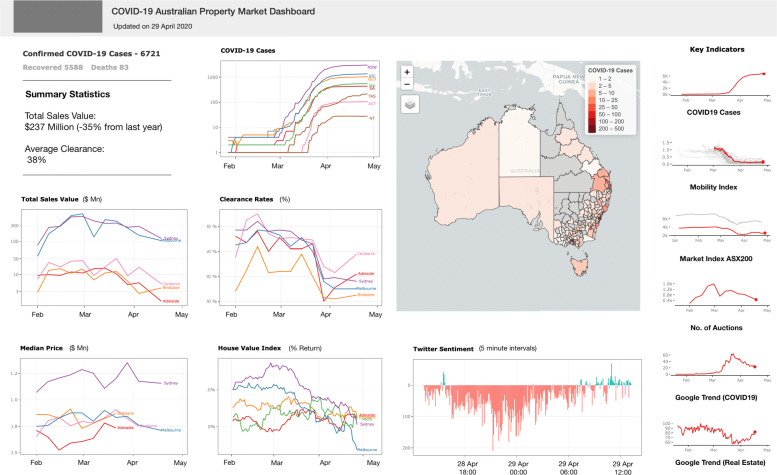
COVID19 Property Dashboard - Australia (Accessed on 22 April 2020)

At the front end, the dashboard is a one-page application available at https://covid19dashboard.be.unsw.edu.au. The data on the dashboard is updated every hour. The user interface of the dashboard has been made interactive using the ‘plotly’ library which lets the users interact with each chart separately where they can ‘zoom’ in and out of the charts temporally in an interactive manner. A snapshot of the dashboard is shown in Fig. 3. The dashboard is responsive and uses a four-column grid layout which collapses into a single column on devices with a narrow screens. The first two columns on the left contain information related to COVID-19 and the property market in Australia, which can be visually contrasted against each other. The third column shows unique information such as the geographic distribution of COVID-19 and public sentiment from Twitter. The rightmost column is reserved for the collection of temporal indicators, which can be compared to each other at a glance.

The dashboard was released to the wider community on 24th April 2020 and has been in use since. It has been covered in news portals such as The Guardian, the Financial Standard etc. and was used by 14000 people across the world with the primary audience based in Australia and the United States. The dashboard was developed to help the government, industry and communities to understand the current impacts of COVID-19 on the Australian property market; it shows the volume and change of these phenomena in tandem and enables users to spot patterns in them. These potential inferences, patterns, and the overall utility of the dashboard are discussed in the next section.

## Discussion

***Insights***

With ‘data-driven science’ emerging as the fourth paradigm of GI science (Gahegan 2020), this dashboard forms the first step in understanding the complexities of real estate markets and their relationships to disruptive events. The dashboard will assist the market stakeholders to better understand, monitor and make more informed decisions concerning property as the COVID-19 pandemic continues to unfold.

When the indicators showing the weekly total value, clearance rate and median price of residential property auctions were plotted against the number of cases of COVID-19 in the corresponding states they show the impact of the pandemic on the market. It is observed that the number of auctions and the clearance rates reduce when there is an increase in the number of COVID-19 cases in the state, which may be due to the restriction of market activities during the lock downs that followed. This is highlighted in the closure of the auction market completely in Melbourne when Victoria experienced a second wave of infections in August 2020. On 22 April 2020, around the peak of the first wave of cases, the dashboard reported that the total volume of sales across Australia that week has been down $237 million compared to the previous year and the clearance rates dropped in all major cities. In contrast to this, the median prices have remained unaffected in 2020, which may be because of the numerous fiscal interventions implemented by the government such as reduction of interest rates, first home owner subsidies and other building incentives.

**Fig. 4:**
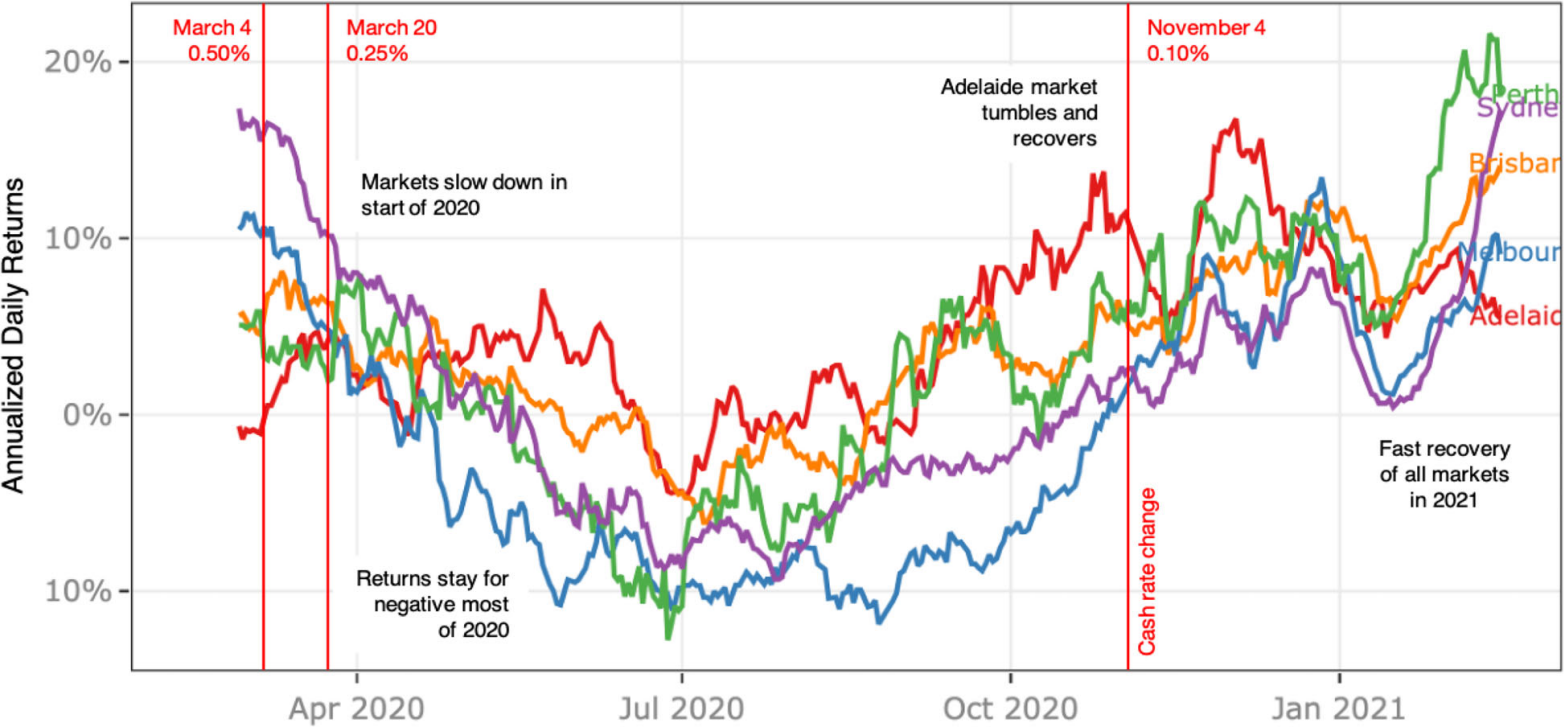
Daily momentum of the House Value Index from March 2020 until Feb 2021 measured as the annualised returns averaged over a 20 day period

The House Value Index indicator (Fig. 4) shows the daily momentum of the market and its reaction to short-term events. We can observe that after the initial fall in February 2020, the annualised return from the daily change of the House Value Index remained negative for most of 2020 behaving similar to an investment in the equity market having an annualised loss of 5–10%. This indicator noticeably captures the impact of the COVID19 cluster in South Australia in November 2020 and the strong upward momentum in markets across Australia since the beginning of 2021. The S&P ASX 200 Index for Real Estate (XRE) also shows a similar trend: the index went down significantly during the first outbreak and has been recovering steadily since, with minimal effects of any subsequent outbreaks.

**Fig. 5:**
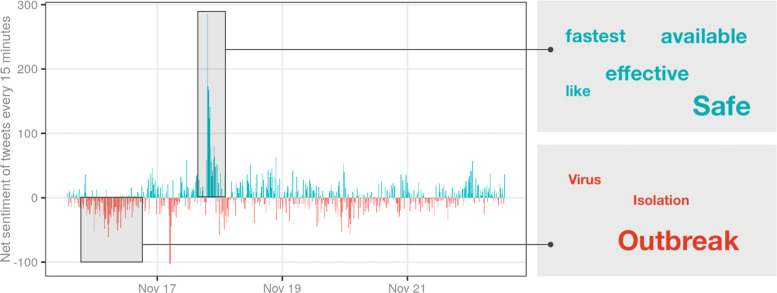
Analysis of tweets collected between 15 and 22nd November 2020 showing the negative and positive sentiments associated with the outbreak in Adelaide and approval of Pfizer vaccines respectively

Out of all the indicators, Twitter sentiment analysis captures the sentiment of the public regarding the COVID-19 with most granularity. Although it is derived from a simplistic classification method, the 15-minute aggregate index reflects the general sentiment and captures reactions to current events and news fairly accurately. For example, Fig. 5 shows the sentiment of tweets collected between 15 November 2020 and 22 November 2020. There is a strong negative sentiment around 16th November, which coincides with the outbreak in Adelaide. Analysis of the words forming this sentiment reveals ‘outbreak’ to be the most used word. Similarly, there is a significant spike in positive sentiment around 18th November, when news relating to Pfizer vaccine broke along with a popular petition to assure disadvantaged members of society’s access to the vaccine. When analysed at the sub-market level, these real-time insights could be of great value to stakeholders in the real estate market (e.g., investors, landlords and renters) during valuation and price discovery. The long-term trends in sentiment and interest can be identified using the Google Search index. The trend for the term ‘real-estate’ in Australia shows the interest in real estate reached the lowest point in the first outbreak in 2020 and has steadily increased since. In contrast, the trend for ‘COVID-19’ shows that public interest has steadily decreased with slight increases around subsequent smaller outbreaks.

**Fig. 6:**
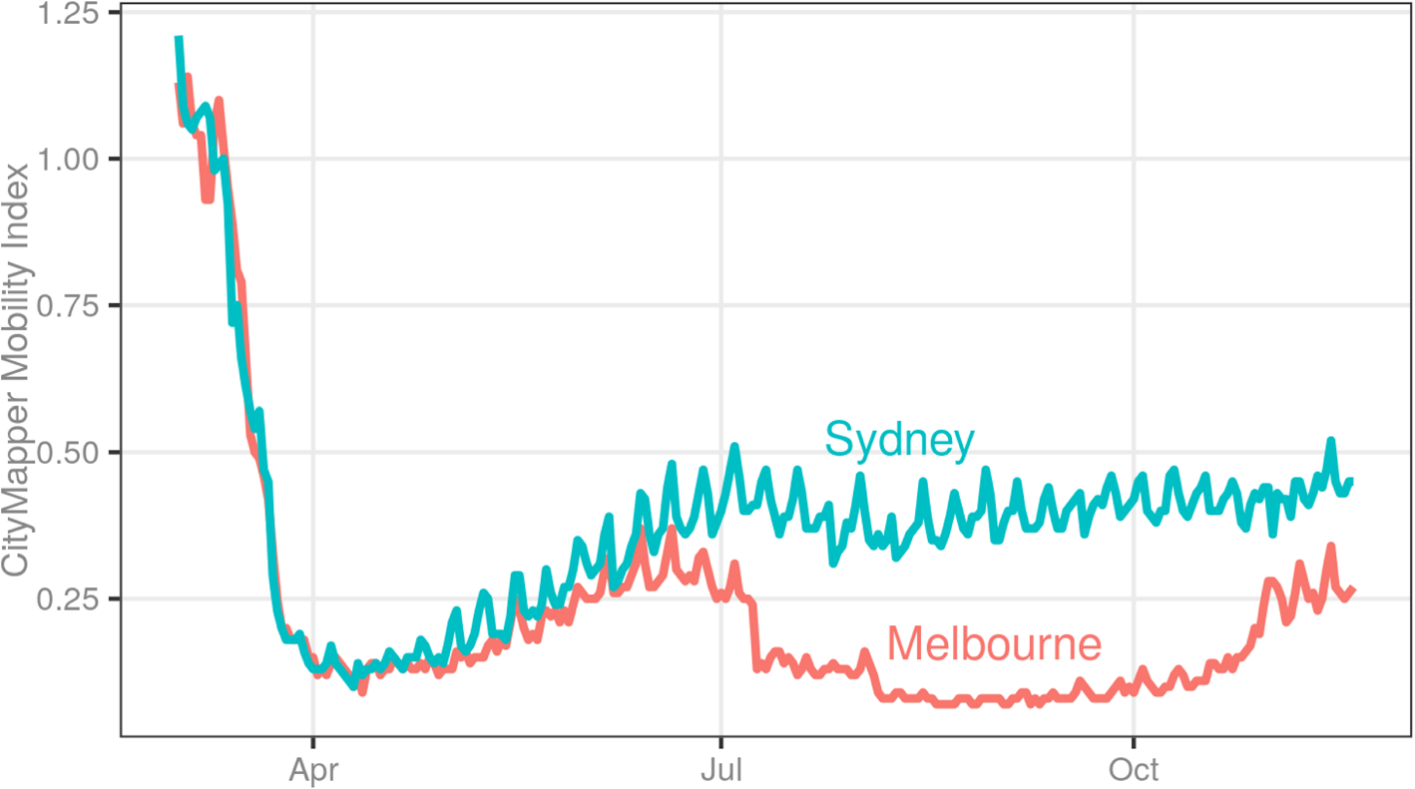
Fall and recovery of the CityMapper mobility index in Melbourne and Sydney

The difference in the spread of the virus between the two major centres of population (Melbourne and Sydney) provides the best opportunity in Australia to see the impact of COVID-19 on these two markets; this can be best observed through the Citymapper Mobility Index shown in Fig. 6. The mobility in both the cities followed the same trajectory at the beginning of the outbreak and during its containment, but the trajectories significantly diverged when the second wave of infections surfaced in Victoria. While Sydney enacted more restrictions and began to cautiously return to normal, Melbourne entered a strict and total lockdown, pushing the mobility to almost zero, and this did not improve until November. Although mobility has since improved, it remains around 40% of the pre-pandemic levels at the time of writing and may take more time to return to normal.

***Usefulness of the dashboard***

The primary aim of the dashboard is to enable the users to monitor the state of the property market and contrast the changes occurring in the market to the ongoing COVID19 pandemic. In this context, the dashboard has proven to be useful in understanding the general movement of the market along with the pandemic indicators. For example, Figs. 4 and 5 show how the user can monitor the changes in house value and public sentiment changes on a continuous basis and potentially inform their decisions based on them. The user can also get an idea of the stage of recovery indirectly using indicators such as ASX200 and Mobility indicators (shown in Fig. 6).

Although this gives us a broad overview, to understand the relationship between these indicators and to develop comprehensive analytical models, a simple Pearson’s correlation analysis between them was conducted. Table 3 shows the correlation coefficients between various indicators used in the dashboard and the corresponding number of COVID-19 cases. We observe very weak correlations between the number of COVID-19 cases and the House Value Index and XRE Index but no significant relationship between the number of cases and the auction values and prices. The most significant correlations are found in Twitter sentiment, XRE and Google Trends, showing that these sentiments accurately reflect the scale and impact of the ongoing pandemic.

**Table 3:** Correlation Coefficients of the Various Indicators to the Corresponding COVID-19 Cases

Indicator	Time	Spatial Aggregation	Pearson’s Coeff.
Auction Value	Weekly	Cities	+0.03
Clearance Rate	Weekly	Cities	-0.19
Median Price	Weekly	Cities	-0.04
House Value Index	Daily	Cities	-0.32
Mobility Index	Daily	Cities	-0.31
ASX 200 XRE	Daily	National	-0.37
Google realestate	Daily	National	-0.32
Google COVID-19	Daily	National	+0.35
Twitter Sentiment	Daily	National	-0.43

We also need to consider the speed of the data and their suitability for use in a real estate market context where the definition of ‘real time’ could be widely different. For example, what would be considered ‘past data’ in social media such as twitter could be considered almost real time in real estate context where transactions can take often weeks to be completed. There is a need to reclassify or reframe what is real-time for real estate markets. From the experience of building this dashboard, we believe that for real estate markets, both spatial and temporal resolution play a significant role in determining the effectiveness of a dashboard. Besides scenarios dealing securitised commodities such as REITs, any data source or indicator with temporal granularity greater than weekly basis could be considered as real time. This understanding can help us avoid situations where higher granularity would require large increase in resources with marginal improvement in quality. The precision and accuracy of the indicators and analytical models in the dashboard need be designed based on the targeted user of the dashboard. For example, visual analytics comparing various data points may be sufficient for a reporting or monitoring use such as this dashboard but for a policy making use we may need much more detailed models and scenario building tools.

***Limitations and challenges***

As discussed in Section 3.1, the biggest challenge we faced was the availability, variety and veracity of the data sources. Most of the data sources concerning the real estate market with high spatio temporal granularity were commercial in nature or contain identifiable personal information hence not relevant to the open data dashboard. A substantial number of available sources are in a format such as PowerBI reports or images that discourage their further use. The data sources from different states varied in their resolution and update frequency, which limited the extent to which we can compare them. This is exacerbated in real estate markets where operational contexts could be significantly different. For example, auctioning of residential properties is much more significant in NSW and VIC compared to other states. Indicators and insights into the rental market have been largely missed in the dashboard due to the lack of timely data. The lexicon-based Twitter sentiment analysis employed is preliminary and cannot capture the nuances of public discourse, such as slang, satire and sarcasm. Compared to the USA and European countries, Australia has had a low number of cases of COVID-19 both in total and as a proportion of population; this could have prevented us from identifying strong patterns relating to the spread of the virus both nationally and regionally. Moreover, along with travel and business restrictions, the Australian Government has introduced various fiscal and social-welfare measures throughout 2020, which could have negated the impact of COVID-19 on the real estate market.

***Future work***

In terms of events disrupting real estate markets, this dashboard focuses on just the COVID-19 pandemic. This could be broadened to make a more flexible monitoring dashboard that can provide insights during similar events in the future. The major portion of such work would be identifying and collecting appropriate data sources and creating indicators that could be substituted instead of COVID-19. One could also focus on rental markets using the data from rental transactions, bond lodgements and short-term leasing platforms such as Airbnb, StayZ and Homestay. Another direction is to develop analytical models using the relationship between the various indicators and the real estate market to quantify the impact in a timely manner. This task is not currently possible to the fullest potential because of the issues discussed earlier; however, it could be attempted again with more granular and reliable data sources. This—along with analytical models—can lead to the development of a predictive models to help estimate and debate the impact and effectiveness of policy decisions and risk reduction methods. With the given volume of information, the sentiment analysis section of the dashboard has great potential for further development as a study in its own right. The sentiment analysis could be improved with the use of advanced natural language processing techniques, and the analysis could be conducted in a range of suburbs and regional markets to understand their dynamics.

## Conclusions

In this paper, we have explored the use of real-time dashboards in monitoring the impact of disruptive events on the real estate market through the case study of COVID-19 in Australia. We have discussed existing literature regarding the characteristics of the real estate market, the impact of disruptive events on it, and city dashboards and their usage in understanding the real estate market. To summarise, the real estate market—which has historically been a conservative, long-term investment market is changing into a faster, more speculative, and more responsive market due to developments in information technology and associated PropTech innovations. As large-scale disruptive events such as economic downturn and natural disasters are occurring more frequently due to the globalizing economy and climate change, there is a clear opportunity for applying the knowledge and expertise in urban planning and building city dashboards to the monitoring of real estate markets.

We aimed to build a COVID-19 property dashboard to help real estate market stakeholders (e.g., investors, buyers, sellers, lenders and policy makers) to monitor and understand the behaviour of the Australian property market during the COVID-19 pandemic. We surveyed the sources of open, usable and comprehensive data concerning real estate markets in Australia and noted the opportunities presented by them. We also highlighted the gaps present in them along with problems in their veracity, spatio-temporal variance and inaccessible formats. Within these limitations, we collected data and designed indicators to track the spread of COVID-19, residential property auctions, house values, market sentiment and mobility across markets in Australia. These indicators were then compiled into a single-page, responsive, interactive dashboard that was implemented and deployed using a micro-services architecture.

The paper has also discussed various insights we could derive from the dashboard by having all these indicators side by side. Some indicators (e.g., auction clearance rates, house values and REIT indices such as XRE) are correlated to the daily new cases of COVID-19 in their corresponding spatial units, while other indicators (e.g., average auction price and total value of auctions) have remained unaffected. The Twitter sentiment index and the mobility index served best in understanding the impact of COVID-19 in the short term, while Google Search trends were found to be useful in the long term. Although numerous relationships present between these indicators were confirmed visually through the dashboard, we found that they were weak when measured through Pearson’s correlation. One of outcomes from the data we observe is that the total auction values and number of COVID19 cases are not significantly correlated in Australia. Although this may appear counter intuitive, our research suggests that the property market in Australia largely avoided any meaningful impact from COVID19 in 2020. This could be attributed to an array of factors such as government interventions such as quantitative easing, home buyer/owner grants and interest rate changes, plasticity of property market in general, etc. There is need for further research regarding this including the data from 2021 & 2022 to identify and quantify the impact of these factors. There is also a need for better infrastructure, standards, and improved frequency of dissemination in real estate data for the sector to transform itself, similar to how urban management and planning transformed under the umbrella of smart cities and research into construction of tools and methodologies for better analytical models. Although, we still remain challenged in realising the real-time digital city discussed by (Kitchin et al. 2015b), we have demonstrated the ability to formulate a Property Dashboard purely driven through open data which can indicate trends in real estate markets, accessibility, mobility, sentiment in the context of the COVID-19 pandemic.

## Data Availability

All datasets used in this research are derived from the open and freely accessible sources as mentioned in the manuscript.
